# Plasma *S*-adenosylmethionine, *DNMT* polymorphisms, and peripheral blood LINE-1 methylation among healthy Chinese adults in Singapore

**DOI:** 10.1186/1471-2407-13-389

**Published:** 2013-08-17

**Authors:** Maki Inoue-Choi, Heather H Nelson, Kim Robien, Erland Arning, Teodoro Bottiglieri, Woon-Puay Koh, Jian-Min Yuan

**Affiliations:** 1 Division of Epidemiology and Community Health, School of Public Health, University of Minnesota, Minneapolis, USA; 2 Masonic Cancer Center, University of Minnesota, Minneapolis, USA; 3 Department of Epidemiology and Biostatistics, George Washington University, Washington, USA; 4 Institute of Metabolic Disease, Baylor Research Institute, Dallas, USA; 5 Saw Swee Hock School of Public Health, National University of Singapore, Singapore, Singapore; 6 Duke-NUS Graduate Medical School, Singapore, Singapore; 7 Cancer Control and Population Sciences, University of Pittsburgh Cancer Institute, Pittsburgh, USA; 8 Department of Epidemiology, University of Pittsburgh Graduate School of Public Health, Pittsburgh, USA

## Abstract

**Background:**

Global hypomethylation of repetitive DNA sequences is believed to occur early in tumorigenesis. There is a great interest in identifying factors that contribute to global DNA hypomethylation and associated cancer risk. We tested the hypothesis that plasma *S*-adenosylmethionine (SAM) level alone or in combination with genetic variation in DNA methyltransferases (*DNMT1*, *DNMT3A* and *DNMT3B*) was associated with global DNA methylation extent at long interspersed nucleotide element-1 (LINE-1) sequences.

**Methods:**

Plasma SAM level and LINE-1 DNA methylation index were measured using stored blood samples collected from 440 healthy Singaporean Chinese adults during 1994-1999. Genetic polymorphisms of 13 loci in *DNMT1*, *DNMT3A* and *DNMT3B* were determined.

**Results:**

LINE-1 methylation index was significantly higher in men than in women (*p* = 0.001). LINE-1 methylation index was positively associated with plasma SAM levels (*p* ≤ 0.01), with a plateau at approximately 78% of LINE-1 methylation index (55 nmol/L plasma SAM) in men and 77% methylation index (50 nmol/L plasma SAM) in women. In men only, the T allele of *DNMT1* rs21124724 was associated with a statistically significantly higher LINE-1 methylation index (*p*_trend_ = 0.001). The *DNMT1* rs2114724 genotype modified the association between plasma SAM and LINE-1 methylation index at low levels of plasma SAM in men.

**Conclusions:**

Circulating SAM level was associated with LINE-1 methylation status among healthy Chinese adults. The *DNMT1* genetic polymorphism may exert a modifying effect on the association between SAM and LINE-1 methylation status in men, especially when plasma SAM level is low. Our findings support a link between plasma SAM and global DNA methylation status at LINE-1 sequences.

## Background

Inter-individual variation in DNA methylation extent has been associated with increased risk for many chronic diseases including cancer [1-4]. Global DNA hypomethylation, the genome-wide loss of methylcytosine, has been observed in malignant and benign tumors and normal tissues surrounding tumors, indicating that global DNA hypomethylation may be one of the early molecular events in carcinogenesis [5-7]. This reduced global DNA methylation impacts repetitive DNA sequences rich in CpG dinucleotides such as long interspersed nucleotide element-1 (LINE-1). Methylation extent at LINE-1 sequences, as a surrogate marker of global DNA methylation status, varies by gender, age and environmental and lifestyle factors [3,8-14].

Methylation of DNA requires the methyl donor *S*-adenosylmethionine (SAM), a key metabolite in one-carbon metabolism (OCM). DNA methyltransferase (DNMT) enzymes transfer a methyl moiety from SAM to the 5th carbon of the cytosine pyrimidine ring at target CpG dinucleotides. A number of epidemiologic studies have evaluated the association between OCM nutrients, such as folate, and the risk of cancer [15-17]. The molecular mechanism for the OCM-cancer association is not completely understood. One hypothesis is that an altered balance in OCM metabolites results in an insufficient supply of methyl moieties for DNMT-catalyzed reactions, resulting in global hypomethylation at DNA sequences [18,19].

In addition to nutrient-related inter-individual variation in the supply of methyl moieties, genetic variation that impacts the activity level of the DNMT enzyme may influence global DNA methylation. DNMT1 is the primary enzyme for maintenance of DNA methylation, whereas DNMT3A and DNMT3B function primarily (but not exclusively) as *de novo* methyltransferases which are responsible for the establishment of DNA methylation patterns in early embryonic development [20-25]. Genetic variation in these *DNMT*s might influence DNA methylation levels and modify the association between SAM status and global DNA methylation.

To advance our understanding of how OCM contributes to cancer susceptibility, there is a need for the establishment of a direct link of plasma SAM and other OCM metabolites to DNA methylation status. In this study, we tested the hypothesis that plasma SAM level, alone or in combination with *DNMT1*, *DNMT3A* and *DNMT3B* genetic variation, was associated with methylation levels at LINE-1 sequences in a healthy human population.

## Methods

### Study subjects

The Singapore Chinese Health Study (SCHS) is a population-based prospective cohort investigation of diet and the risk for cancer and other chronic diseases. The detailed study design of the SCHS has been described previously [26]. In brief, Chinese men and women aged 45 – 74 years who were permanent residents in Singapore were invited to participate in the study from April 1993 through December 1998. A total of 63,257 participants (85% of the eligible individuals) were enrolled. Baseline information including demographic and lifestyle factors, medical history, family history of cancer and usual dietary intake was collected through in-person interviews at recruitment.

A 3% random sample of the cohort was contacted to donate blood or urine samples starting in 1994. By the end of cohort participant enrollment in 1999, 1,194 subjects donated blood (n = 906) or buccal cells (n = 288). Two 10-mL tubes of blood were drawn from each cohort participant and immediately placed on ice during the transportation to the National University of Singapore. At the laboratory, one tube blood was processed and separated into plasma, buffy coat, and red blood cells, and the other for serum. All blood components were stored in a liquid nitrogen tank at -180°C until 2001, when they were moved to -80°C freezers for long term storage. The present study was based on the subjects who were included in a nested study on plasma homocysteine initiated in 1996-1997 [26]. By that time, 509 subjects had donated blood samples. These subjects had somewhat higher education, lower prevalence of smoking, and higher prevalence of alcohol intake than the overall SCHS participants, but otherwise were comparable to the whole cohort participants in terms of age, height, body weight, and BMI. This study was approved by the Institutional Review Boards at the University of Minnesota and the National University of Singapore. Prior to study participation, written informed consent was obtained from participants.

### SNP selection and genotype determinations

We selected common single nucleotide polymorphisms (SNPs) of *DNMT1*, *DNMT3A* and *DNMT3B* with a minor allele frequency (MAF) ≥20%, given the relatively small sample size of the present study. Six SNPs were chosen based on their reported association with cancer [27,28]: rs2114724, rs2241531, rs1863771, rs1699593, and rs75616428 for *DNMT1* and rs1550117 for *DNMT3A*. Additional 12 SNPs were selected for haplotype tagging using Han Chinese (CHB) data in the International HapMap Project database (Tagger Pairwise method, HapMap Data Rel 27 Phase II + III, Feb09, on NCBI B36 assembly, dbSNP b126): rs2228611, rs2288350 and rs7253062 for *DNMT1*; rs6722613, rs7575625, rs7581217, rs7587636, rs12987326, rs12999687, rs13036246 and rs34048824 for *DNMT3A*; and rs2424908 and rs6141813 for *DNMT3B*. One SNP of *DNMT1* (rs1863771) failed in the Sequenom assay design, and 2 SNPs of *DNMT1* (rs1699593 and rs75616428) did not display genetic variation. Two SNPs of *DNMT3A* (rs12987326 and rs12999687) were excluded from the analysis because they were not in Hardy-Weinberg equilibrium (*p* <0.05). As a result, we report a total of 13 SNPs (4 SNPs of *DNMT1*, 7 SNPs of *DNMT3A*, and 2 SNPs of *DNMT3B*).

DNA was extracted from stored buffy coats using a Qiagen QIAmp 96 DNA Blood Kit (Qiagen Inc.), and genotype determination was performed in multiplex using the Sequenom MALDI-TOF mass spectrometry system (Sequenom Inc.) by the University of Minnesota BioMedical Genomics Center (BMGC). Each 96-well plate contained positive and negative controls.

### Laboratory measurements

We used stable-isotope dilution liquid chromatography-electrospray ionization (ESI) tandem mass spectrometry (LC-ESI-MS/MS) to determine SAM and *S*-adenosylhomocysteine (SAH) concentrations in plasma. Calibrators and internal standards (^2^H_3_-SAM and ^2^H_4_-SAH) were included in each analytical run for calibration. One-mM stock solutions of each standard were diluted in distilled water to perform a 5-point calibration curve (Table 1). Sample preparation involved ultrafiltration utilizing microcentrifugal filter units, Microcon YM-10, 10 kDa NMWL (Millipore). Samples were prepared by the addition of 100 μL mobile phase A containing 10 – 50 μmol/L labeled-isotope internal standards to 30 μl of standard or plasma. Sample filtrate was transferred to a microtiter plate for analysis. Chromatographic separation was achieved on an EZ-faast 250 × 2.0 mm 4 μ AAA-MS analytical column (Phenomenex) maintained at 36°C at a flow of 250 μL/min with a binary gradient with a total run time of 12 minutes. Solvents for HPLC were: (A) 4 mM ammonium acetate, 0.1% formic acid and 0.1% heptafluorobutyric acid (pH = 2.5); and (B) 100% methanol and 0.1% formic acid. The compounds were detected by multiple reaction monitoring (MRM) using positive-ESI. Sample separation and injection was performed by a Shimadzu Prominence LC System interfaced with a 4000 Q TRAP® LC-MS/MS (ABSciex). All data were collected using Analyst software version 1.4.2. The inter-assay precision (CV%) was 6.2 – 9.2% for SAM and 5.9 – 10.4% for SAH.

**Table 1:** Mass transitions and method statistics for the determination of plasma SAM and SAH

							**Inter-assay precision**	
**Analyte**	**Analyte**	**Labeled**	**Labeled isotope**	**Retention time**	**LOQ**	**Calibration**	**Level 1**	**Level 2**
	**MRM (m/z)**	**isotope**	**MRM (m/z)**	**(minutes)**	**(nmol/L)**	**curve**	**(CV%)**	**(CV%)**
SAM	399 → 250	^2^H_3_-SAM	402 → 250	7.1	5	400 – 25 nmol/L	9.2	6.2
SAH	385 → 136	^2^H_4_-SAH	389 → 138	6.8	5	400 – 25 nmol/L	10.4	5.9

LINE-1 DNA methylation was quantified using the quantitative bisulfite PCR pyrosequencing method developed by Yang *et al*[29]. Genomic DNA from peripheral lymphocytes was sodium bisulfite treated using the EZ-96 DNA Methylation Kit, converting non-methylated cytosine residues into uracil, according to the manufacturer’s protocol (Zymo Research). Bisulfite converted DNA was PCR amplified using Hotstar Taq Polymerase (Qiagen Inc.). For pyrosequencing, the PCR product was purified using a biotin-labeled primer and Streptavadin Sepharose beads (GE Healthcare). The bead immobilized PCR product was purified, washed, denatured using a NaOH solution, and washed again using the Pyrosequencing Vacuum Prep Tool (Pyrosequencing, Inc.). PCR amplifications were done in triplicate and the extent of methyl cytosine relative to the total cytosine and thymine (%) at each of 4 CpG sites was measured. The average of methylation across the four CpG sites was computed for each replicate, and the average of three replicate measurements of LINE-1 DNA methylation was used as LINE-1 methylation index (%) for each sample in the analysis.

### Statistical analysis

Twenty-nine subjects with missing values of LINE-1 methylation index were excluded. Of the remaining 480 subjects, 16 subjects who had missing values (n = 14) or extremely high values (n = 2) of plasma SAM level, and 24 subjects whose serum creatinine values were missing (n = 23) or extremely high (n = 1) were also excluded. As a result, a total of 440 subjects were included in the analysis of plasma SAM level and LINE-1 methylation index. In addition, 8 subjects with missing values for 2 or more genotypes were omitted in the analysis of *DNMT1*, *DNMT3A*, and *DNMT3B* genotypes and haplotypes and LINE-1 methylation index.

All analyses were conducted in men and women separately, given the difference in LINE-1 methylation index between sexes [30-32]. Spline curves were created to visualize the association between plasma SAM and LINE-1 methylation index and to determine acut-off value of low LINE-1 methylation index. Using cut points identified in the spline curves, LINE-1 methylation index was compared across plasma SAM categories (<55 nmol/L and ≥55 nmol/L for men and <50 nmol/L, 50 – 90 nmol/L and ≥90 nmol/L for women) using multiple linear regression modeling with age at blood draw and serum creatinine level as covariates. We adjusted the analysis for serum creatinine level because it has been associated with the SAM-SAH ratio [33,34], and was associated with both plasma SAM level and LINE-1 methylation index in our study population. Similarly, LINE-1 methylation index was compared across genotypes of each *DNMT*. Haplotypes with a 5% or more frequency were constructed. Logistic regression modeling was used to calculate the odds of being in the low LINE-1 methylation index group (<78% for men and <77% for women) for each haplotype relative to the most frequently observed haplotype. A series of spline curves for the relationship between plasma SAM and LINE-1 methylation index by *DNMT* genotypes were created to evaluate a potential modifying effect of *DNMT* genotypes on the association between plasma SAM level and LINE-1 methylation index.

Haplotype analysis was performed using R version 2.13.2, haplostat package. All other analyses were conducted using SAS version 9.2 (SAS Institute Inc.). All reported *p* values are two-sided, and those that were < 0.05 were considered to be statistically significant. A significance level was adjusted as *p* < 0.0038 (0.05/13 SNPs) for multiple testing of *DNMT* SNPs and LINE-1 methylation index using the Bonferroni correction method.

## Results

The average age of the 440 study subjects was 58.1 y (standard deviation (SD), 7.8 y; range, 46 – 77 y). Approximately 72% of the study subjects had body mass index (BMI) below 24 while only 4% had BMI of 28 or above. Men had slightly higher education level than women; 40% of men had secondary school or some college education, while 25% of women attained the same level of education. About 56% of men and 7% of women were past or current smokers. Approximately 30% of men and 7% of women consumed alcoholic beverages on a regular basis. The mean plasma SAM level was higher in men (69.7 nmol/L; range, 26.6 – 156.0 nmol/L) than women (63.3 nmol/L; range, 17.2 – 149.0 nmol/L); however, the geometric mean of plasma SAM level after adjustment for age at blood draw and serum creatinine level was comparable (63.3 nmol/L in men and 63.9 nmol/L in women). Plasma SAM level was higher with higher BMI and never smoking status in men, and with older age in women in our study population [35]. Plasma SAM level was not different by education level, alcohol intake, and menopausal status (in women). Plasma SAM level was not associated with methylenetetrahydrofolate reductase (*MTHFR*) C677T genotype or plasma total homocysteine (tHcy) level [35]. Comorbid conditions that may affect the absorption of nutrients in OCM (stomach or duodenum ulcer, partial removal of stomach, and polyps of intestine) were not associated with plasma SAM level or LINE-1 methylation index (data not shown).

LINE-1 methylation index was normally distributed ranging from 68.7% to 83.4% with an average of 77.7% in all subjects. Table 2 shows the mean levels of LINE-1 methylation index across different groups of age, BMI, level of education, smoking and alcohol consumption for men and women separately. The mean LINE-1 methylation index was statistically significantly higher in men (78.1%) than in women (77.3%) (*p* = 0.001). LINE-1 methylation index was positively associated with age in men, but not in women. High BMI was associated with slightly lower LINE-1 methylation index in women only. There was no statistically significant difference in LINE-1 methylation index by level of education, smoking status or alcohol consumption, in both sexes.

**Table 2:** LINE-1 methylation index (%) by demographic and lifestyle factors in the Singapore Chinese health study

	**Men**			**Women**		
	**n**	**Mean (95% CI)**^**a**^	***p***_**trend**_	**n**	**Mean (95% CI)**^**a**^	***p***_**trend**_
LINE-1 methylation (%)	192	78.1 (77.8 – 78.4)^b^	-	248	77.3 (77.1 – 77.6)^b^	-
Age						
<50	25	77.8 (77.0 – 78.7)	0.04	41	77.0 (76.3 – 77.8)	0.82
50 – 59	79	77.8 (77.3 – 78.3)		123	77.6 (77.1 – 78.0)	
60 – 69	71	78.2 (77.7 – 78.7)		55	77.2 (76.5 – 77.8)	
≥70	17	79.3 (78.2 – 80.3)		29	77.1 (76.3 – 78.0)	
BMI						
<20	23	78.3 (77.4 – 79.2)	0.31	38	77.3 (76.5 – 78.1)	0.08
20 – <24	112	77.8 (77.4 – 78.3)		143	77.6 (77.2 – 78.0)	
24 – <28	49	78.4 (77.7 – 79.0)		56	76.9 (76.3 – 77.5)	
≥28	8	78.9 (77.4 – 80.4)		11	76.1 (74.7 – 77.6)	
Level of education						
No formal school	27	77.8 (76.9 – 78.6)	0.28	93	77.0 (76.5 – 77.6)	0.19
Primary school	89	77.9 (77.5 – 78.4)		94	77.5 (77.0 – 78.0)	
Secondary school	53	78.5 (77.9 – 79.1)		52	77.6 (76.9 – 78.3)	
Some college or above	23	78.2 (77.2 – 79.1)		9	77.6 (76.0 – 79.2)	
Smoking status						
Never	84	78.2 (77.7 – 78.7)	0.60	234	77.4 (77.1 – 77.7)	0.21
Past	49	78.0 (77.4 – 78.7)		2	78.5 (75.1 – 81.9)	
Current	59	78.0 (77.4 – 78.6)		12	76.4 (74.9 – 77.8)	
Alcohol intake						
None	135	78.1 (77.7 – 78.5)	0.98	231	77.4 (77.1 – 77.7)	0.12
<7 drinks/week	42	78.1 (77.4 – 78.8)		13	77.1 (75.7 – 78.4)	
≥7 drinks/week	15	78.0 (76.9 – 79.2)		4	75.3 (72.9 – 77.7)	

^a^Adjusted for age at blood draw.

^b^*p* for difference between men and women was 0.001.

**Figure 1:**
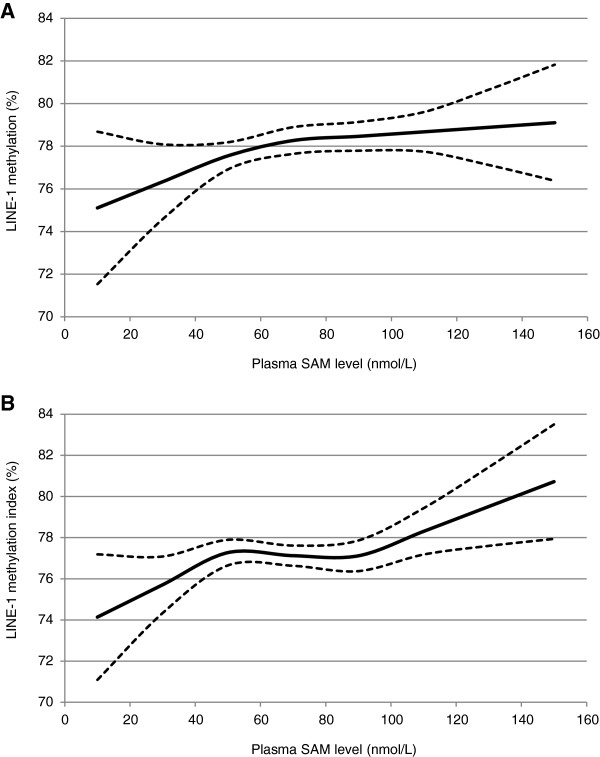
**LINE-1 methylation and plasma SAM level in men (A) and women (B).** * Adjusted for age at blood draw and serum creatinine level. ** Solid lines: mean LINE-1 methylation index corresponding to the mean plasma SAM level; dashed lines: upper and lower 95% confidence intervals.

Figure 1 shows spline curves of LINE-1 methylation index by plasma SAM level. LINE-1 methylation index was positively associated with plasma SAM level, with a plateau at approximately 78% methylation (55 nmol/L plasma SAM) in men and 77% methylation (50 nmol/L plasma SAM) in women, with a second rise in methylation at 90 nmol/L plasma SAM for women.

Men with plasma SAM level at 55 nmol/L or above had statistically significantly higher LINE-1 methylation index than men with low plasma SAM level (*p* = 0.01) (Table 3). Similarly, in women, there was a statistically significant positive trend in LINE-1 methylation index across the three categories of plasma SAM level (*p*_trend_ = 0.005).

**Table 3:** LINE-1 methylation index (%) by plasma SAM level in the Singapore Chinese health study

**Men**				**Women**			
**SAM (nmol/L)**	**n**	**LINE-1 methylation**^**a**^	***p***	**SAM (nmol/L)**	**n**	**LINE-1 methylation**^**a**^	***p***_***trend***_
< 55	38	77.3 (76.6 – 78.0)	0.01	< 50	48	76.6 (75.9 – 77.2)	0.005
≥ 55	154	78.3 (77.9 – 78.6)		50 – < 90	180	77.5 (77.1 – 77.8)^b^	
				≥ 90	20	78.3 (77.2 – 79.4)^c^	

^a^Mean (95% CI) adjusted for age at blood draw and serum creatinine level.

^b^*p* = 0.02 compared to plasma SAM levels <50 nmol/L.

^c^*p* = 0.01 compared to plasma SAM levels <50 nmol/L.

The associations between *DNMT* SNPs and LINE-1 methylation index are presented in Table 4. There was no statistically significant association between any of the 13 SNPs and LINE-1 methylation index in women. In men, there was a statistically significant positive association between the number of variant allele (T) of the *DNMT1* rs2114724 and LINE-1 methylation index (*p*_trend_ = 0.001). In contrast, the number of variant allele (T) of the *DNMT3A* rs758127 was inversely associated with LINE-1 methylation index (*p*_trend_ = 0.008). However, this association did not reach the significance level after taking into account for multiple comparisons. LINE-1 methylation index was not associated with any other SNPs examined (Table 4) or haplotypes of individual *DNMT*s (data not shown).

**Table 4:** **LINE-1 methylation index (%) by genotypes of *DNMT1*, *DNMT3A *and *DNMT3B *in the Singapore Chinese health study**

	**Men**				**Women**			
	**n**^**a**^	**Mean (95% CI)**^**b**^	***p***	***p***_**trend**_	**n**^**a**^	**Mean (95% CI)**^**b**^	***p***	***p***_**trend**_
***DNMT1***								
rs2114724								
CC	90	77.7 (77.2 – 78.1)		0.001	126	77.2 (76.8 – 77.6)		0.38
CT	71	78.3 (77.8 – 78.8)	0.06		98	77.6 (77.1 – 78.1)	0.25	
TT	21	79.3 (78.4 – 80.3)	0.002		20	77.4 (76.3 – 78.5)	0.75	
rs2241531								
GG	44	77.9 (77.2 – 78.5)		0.11	61	77.1 (76.5 – 77.7)		0.71
CG	94	78.1 (77.6 – 78.5)	0.60		121	77.5 (77.1 – 78.0)	0.29	
CC	46	78.6 (78.0 – 79.2)	0.11		63	77.3 (76.7 – 77.9)	0.70	
rs2288350								
CC	55	78.5 (77.9 – 79.1)		0.05	74	77.4 (76.8 – 78.0)		0.57
CT	93	78.1 (77.6 – 78.6)	0.31		115	77.5 (77.0 – 77.9)	0.85	
TT	38	77.6 (76.9 – 78.3)	0.05		55	77.1 (76.5 – 77.8)	0.53	
rs7253062								
GG	109	78.3 (77.9 – 78.7)		0.12	149	77.3 (76.9 – 77.7)		0.92
GA	72	77.8 (77.3 – 78.3)	0.13		83	77.5 (76.9 – 78.0)	0.71	
AA	4	77.6 (75.4 – 79.8)	0.54		11	77.1 (75.7 – 78.6)	0.78	
***DNMT3A***								
rs1550117								
CC	113	78.3 (77.8 – 78.7)		0.10	146	77.5 (77.1 – 77.9)		0.30
CT	58	78.0 (77.4 – 78.5)	0.39		92	77.1 (76.6 – 77.6)	0.24	
TT	15	77.3 (76.2 – 78.4)	0.12		8	77.3 (75.6 – 79.0)	0.83	
rs6722613								
GG	93	78.0 (77.6 – 78.5)		0.87	101	77.4 (76.9 – 77.9)		0.69
GA	69	78.2 (77.7 – 78.8)	0.58		112	77.4 (76.9 – 77.8)	0.93	
AA	24	78.0 (77.1 – 78.9)	0.93		33	77.2 (76.3 – 78.0)	0.65	
rs7575625								
AA	115	78.2 (77.8 – 78.6)		0.29	148	77.3 (76.9 – 77.7)		0.79
AG	59	78.2 (77.7 – 78.8)	0.86		83	77.3 (76.8 – 77.8)	0.98	
GG	11	77.0 (75.7 – 78.2)	0.08		15	77.6 (76.4 – 78.9)	0.66	
rs7581217								
CC	59	78.5 (78.0 – 79.1)		0.008	83	77.5 (77.0 – 78.0)		0.92
CT	94	78.1 (77.7 – 78.6)	0.27		114	77.1 (76.7 – 77.6)	0.26	
TT	33	77.2 (76.4 – 77.8)	0.006		49	77.6 (76.9 – 78.3)	0.90	
rs7587636								
GG	95	78.1 (77.6 – 78.5)		0.89	116	77.2 (76.8 – 77.7)		0.95
GA	68	78.2 (77.6 – 78.7)	0.86		103	77.6 (77.1 – 78.0)	0.32	
AA	22	78.1 (77.2 – 79.1)	0.94		27	77.0 (76.0 – 77.9)	0.57	
rs13036246								
CC	102	78.0 (77.5 – 78.4)		0.26	129	77.5 (77.1 – 77.9)		0.14
CT	68	78.2 (77.7 – 78.7)	0.47		106	77.3 (76.8 – 77.7)	0.48	
TT	15	78.6 (77.5 – 79.7)	0.31		10	76.1 (74.6 – 77.6)	0.08	
rs34048824								
TT	116	78.1 (77.7 – 78.5)		0.61	150	77.3 (76.9 – 77.7)		0.70
TC	58	78.3 (77.8 – 78.9)	0.59		77	77.3 (76.8 – 77.9)	0.99	
CC	10	77.1 (75.8 – 78.5)	0.18		18	77.7 (76.5 – 78.8)	0.57	
***DNMT3B***								
rs2424908								
CC	61	77.9 (77.3 – 78.4)		0.83	88	77.4 (76.9 – 77.9)		0.88
CT	79	78.4 (77.9 – 78.9)	0.18		121	77.3 (76.9 – 77.8)	0.88	
TT	44	77.9 (77.2 – 78.6)	0.96		37	77.3 (76.5 – 78.1)	0.91	
rs6141813								
AA	82	78.0 (77.5 – 78.5)		0.76	103	77.2 (76.8 – 77.7)		0.74
AG	73	78.3 (77.8 – 78.8)	0.35		115	77.4 (77.0 – 77.9)	0.56	
GG	30	78.0 (77.2 – 78.8)	0.99		27	77.3 (76.3 – 78.2)	0.94	

^a^Subjects with missing data on a genotype of each SNP were excluded from an analysis.

^b^Adjusted for age at blood draw.

**Figure 2:**
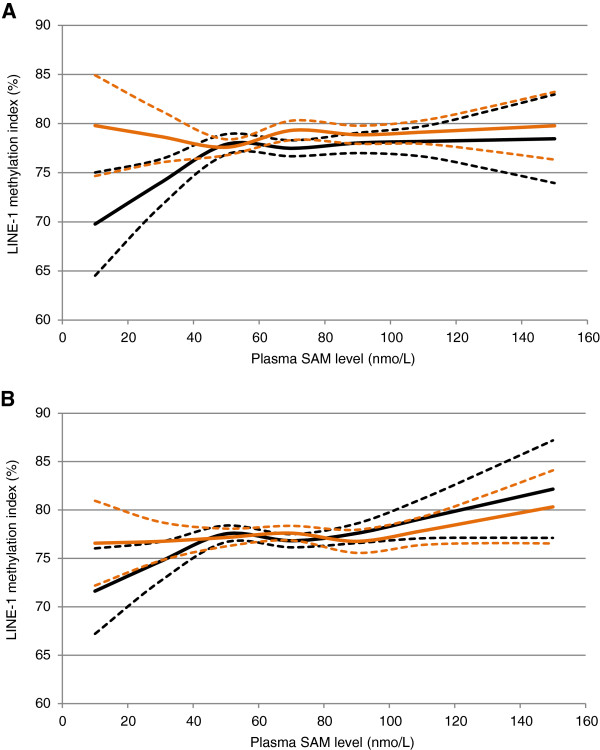
LINE-1 methylation and plasma SAM by *DNMT1 *rs2114724 genotype in men (A) and women (B). ^*^ Adjusted for age at blood draw and serum creatinine level. ^**^ Black line: the wild type genotype (CC) (n = 90 for men and n = 126 for women); red line: variant genotypes (CT and TT) (n = 93 for men and n = 118 for women); dashed lines: upper and lower 95% confidence intervals.

Finally, we examined whether the *DNMT1* rs2114724 genotype modified the association between plasma SAM level and LINE-1 methylation index (Figure 2). Below the threshold of 78% LINE-1 methylation index (55 nmol/L plasma SAM), plasma SAM level was positively associated with LINE-1 methylation index in men carrying the CC genotype of *DNMT1* rs2114724, while there was no association between plasma SAM and LINE-1 methylation index in men possessing the CT or TT genotype. Among men carrying variant genotypes, LINE-1 methylation index was constantly at or above 78% regardless of plasma SAM level. Such an effect modification by the *DNMT1* rs2114724 genotype was not observed among women. Other SNPs of *DNMT1*, *DNMT3A* and *DNMT3B* did not modify the SAM-LINE-1 methylation association (data not shown).

## Discussion

Inter-individual variation in DNA methylation, specifically global hypomethylation of DNA in circulating lymphocytes, has been associated with risk for several diseases including cancer. Our results showed that LINE-1 methylation index, as a surrogate marker of global DNA methylation status, was positively associated with plasma SAM level, and this dose-response relationship plateaued at approximately 78% methylation for men (at 55 nmol/L SAM) and 77% methylation for women (at 50 nmo/L SAM). These findings support the hypothesis that circulating SAM level influences global DNA methylation. In addition, we have described genetic variation in *DNMT1* that influences LINE-1 methylation and possibly modifies the association between plasma SAM and LINE-1 methylation in men.

One possible explanation for the observed plateau in LINE-1 methylation with increasing SAM is an active inhibition of DNA methylation by SAH. After providing a methyl moiety for methylation, SAM is converted to SAH, an established competitive inhibitor of DNMTs [36-38]. We have previously demonstrated a significant positive association between plasma levels of SAM and SAH in this study population [35]. However, neither plasma SAH level nor the SAM:SAH ratio was associated with LINE-1 methylation index (data not shown). Future studies are needed to firmly establish the presence of this plateau and the biologic drivers of its formation.

Methyltransferase enzymes are necessary to establish and maintain the epigenome, and in mice, *DNMT1* mutation results in global DNA hypomethylation [39,40]. DNMT1 enzyme is expressed in proliferating cells and somatic tissues with a major function as a maintenance methyltransferase, copying the existing parental-strand DNA methylation pattern onto the daughter strand after DNA replication [20,21]. In contrast, DNMT3A and DNMT3B are enzymes that are highly expressed in embryonic cells, early embryos, and germ cells, where *de novo* DNA methylation occurs, but are downregulated in somatic tissues [41].

One striking observation across studies is consistently lower LINE-1 methylation among women [11,30,31]. The mechanism of this sex difference is not established. However, LINE-1 elements are believed to be involved in X chromosome inactivation [42] and the X chromosome is enriched nearly 2-fold for LINE-1 elements compared with autosomes [43]. A recent study reported that the major group of LINE-1 promoter regions were significantly hypomethylated on inactive X chromosomes compared with active X chromosomes [30]. They also reported that while total LINE-1 methylation extent was lower in women compared with men, methylation at specific LINE-1 elements on autosomes was not differentially methylated by gender. This differential biologic pressure for LINE-1 methylation on the sex chromosome may underlie the gender difference observed in our and other epidemiologic studies.

The present study has several limitations. One concern is the possible degradation of SAM in stored plasma samples during prolonged storage. The median plasma SAM level in the present study (63.3 nmol/L; interquartile range, 54.5 – 76.0 nmol/L) was somewhat lower compared with the reference values in populations in Europe (70 – 128 nmol/L) [44]. However, the wide range of plasma SAM value (50 – 150 nmol/L) in various studies might be due to different study populations and laboratory methods [36,45-50]. In our study population, we measured plasma tHcy level in the same subjects at two different times with more than 10 years apart (in 1996 – 1997 and in 2010). High correlation between the two tHcy measurements (r, 0.70) indicates that the degradation of Hcy, and possibly SAM, might have been minimal. Furthermore, degradation of plasma SAM would not result in the observed associations, given that it occurred in a non-differential manner. LINE-1 is the most common family of retrotransposons in the human genome and accounts for at least 17% of human DNA, but methylation extent at LINE-1 is not a direct measurement of global DNA methylation [51,52]. LINE-1 is dispersed across the genome; however, because there is a mix of both active and inactive LINE-1 elements present in the genome, it cannot be viewed as either a passive dosimeter of methylation processes or a reflection of methylation processes at active chromatin. Therefore, we are limited in our interpretation of LINE-1 methylation index in examining exposures and disease risk. Whether the methylation status of other surrogate markers of global DNA methylation across the genome are associated with plasma SAM levels remains to be investigated in future studies. Another limitation of the present study is potentially limited variability in LINE-1 methylation because all of the study subjects were cancer-free, healthy individuals. A relatively small sample size is also a limitation. We did not have enough power to test an interaction between plasma SAM, *DNMT* genetic polymorphisms and the LINE-1 methylation index. Lastly, our study did not investigate other factors that may affect the SAM-SAH ratio, such as glucose-6-phosphate dehydrogenase deficiency, folate transport deficiency, and other genetic factors (e.g., glycine *N*-methyltransferase: *GNMT*), as cofounders. Nonetheless, our data suggest that genetic variation in *DNMT* may influence LINE-1 methylation index in peripheral blood in a Chinese population in South Asia.

## Conclusion

Our findings provide supporting data for the association between circulating SAM level and DNA methylation at LINE-1 sequences in peripheral blood in healthy Chinese men. While preliminary, our data also suggest that this link between plasma SAM level and global DNA methylation at LINE-1 sequences in men may be modified by *DNMT1* genetic variation.

## Abbreviations

BMI: Body mass index; DNMT: DNA methyltransferase; ESI: Electrospray ionization; HPLC: High performance liquid chromatography; LINE-1: Long interspersed nucleotide element-1; MAF: Minor allele frequency; MS: Mass spectrometry; OCM: One-carbon metabolism; PCR: Polymerase chain reaction; SAH: *S*-adenosylhomocysteine; SAM: *S*-adenosylmethionine; SCHS: Singapore Chinese Health Study; SNP: Single nucleotide polymorphism.

## Competing interests

None of the authors had a conflict of interest to declare.

## Authors’ contributions

MI-C participated in study design and concept of the research, performed statistical analysis, participated in data interpretation, and drafted the manuscript. HHN participated in study design and concept of the research, LINE-1 methylation determination, and data analysis and interpretation, and helped to draft the manuscript. KR participated in study design and concept of the research. EA performed assays for plasma levels of SAM and SAH and helped to draft the manuscript. TB performed assays for plasma levels of SAM and SAH and participated in data interpretation. W-PK participated in acquisition of data. J-MY participated in study concept, design and obtaining funding to support the parent cohort of the present project, acquisition of data, study design and concept of the research, data interpretation, and helped to draft the manuscript. All authors read and approved the final manuscript.

## Pre-publication history

The pre-publication history for this paper can be accessed here:

http://www.biomedcentral.com/1471-2407/13/389/prepub

## References

[B1] Ehrlich M DNA methylation in cancer: too much, but also too little Oncogene 2002 21 35 5400 5413 10.1038/sj.onc.1205651 12154403

[B2] Esteller M Corn PG Baylin SB Herman JG A gene hypermethylation profile of human cancer Cancer Res 2001 61 8 3225 3229 11309270

[B3] Zhu ZZ Sparrow D Hou L Tarantini L Bollati V Litonjua AA Zanobetti A Vokonas P Wright RO Baccarelli A Repetitive element hypomethylation in blood leukocyte DNA and cancer incidence, prevalence, and mortality in elderly individuals: the Normative Aging Study Cancer Causes Control 2011 22 3 437 447 10.1007/s10552-010-9715-2 21188491 PMC3752839

[B4] Liao LM Brennan P Van Bemmel DM Zaridze D Matveev V Janout V Kollarova H Bencko V Navratilova M Szeszenia-Dabrowska N LINE-1 methylation levels in leukocyte DNA and risk of renal cell cancer PLoS One 2011 6 11 e27361 10.1371/journal.pone.0027361 22076155 PMC3208631

[B5] Goelz SE Vogelstein B Hamilton SR Feinberg AP Hypomethylation of DNA from benign and malignant human colon neoplasms Science 1985 228 4696 187 190 10.1126/science.2579435 2579435

[B6] Suter CM Martin DI Ward RL Hypomethylation of L1 retrotransposons in colorectal cancer and adjacent normal tissue Int J Colorectal Dis 2004 19 2 95 101 10.1007/s00384-003-0539-3 14534800

[B7] Gama-Sosa MA Slagel VA Trewyn RW Oxenhandler R Kuo KC Gehrke CW Ehrlich M The 5-methylcytosine content of DNA from human tumors Nucleic Acids Res 1983 11 19 6883 6894 10.1093/nar/11.19.6883 6314264 PMC326421

[B8] Cash HL Tao L Yuan JM Marsit CJ Houseman EA Xiang YB Gao YT Nelson HH Kelsey KT LINE-1 hypomethylation is associated with bladder cancer risk among nonsmoking Chinese Int J Cancer 2012 130 5 1151 1159 10.1002/ijc.26098 21445976 PMC3208798

[B9] Wilhelm CS Kelsey KT Butler R Plaza S Gagne L Zens MS Andrew AS Morris S Nelson HH Schned AR Implications of LINE1 methylation for bladder cancer risk in women Clin Cancer Res 2010 16 5 1682 1689 10.1158/1078-0432.CCR-09-2983 20179218 PMC2831156

[B10] Bollati V Baccarelli A Hou L Bonzini M Fustinoni S Cavallo D Byun HM Jiang J Marinelli B Pesatori AC Changes in DNA methylation patterns in subjects exposed to low-dose benzene Cancer Res 2007 67 3 876 880 10.1158/0008-5472.CAN-06-2995 17283117

[B11] Zhu ZZ Hou L Bollati V Tarantini L Marinelli B Cantone L Yang AS Vokonas P Lissowska J Fustinoni S Predictors of global methylation levels in blood DNA of healthy subjects: a combined analysis Int J Epidemiol 2010 41 1 126 139 20846947 10.1093/ije/dyq154 PMC3304518

[B12] Zhang FF Morabia A Carroll J Gonzalez K Fulda K Kaur M Vishwanatha JK Santella RM Cardarelli R Dietary patterns are associated with levels of global genomic DNA methylation in a cancer-free population J Nutr 2011 141 6 1165 1171 10.3945/jn.110.134536 21525250 PMC3095144

[B13] Zhang FF Santella RM Wolff M Kappil MA Markowitz SB Morabia A White blood cell global methylation and IL-6 promoter methylation in association with diet and lifestyle risk factors in a cancer-free population Epigenetics 2012 7 6 606 614 10.4161/epi.20236 22531363 PMC3398989

[B14] Rusiecki JA Baccarelli A Bollati V Tarantini L Moore LE Bonefeld-Jorgensen EC Global DNA hypomethylation is associated with high serum-persistent organic pollutants in Greenlandic Inuit Environ Health Perspect 2008 116 11 1547 1552 10.1289/ehp.11338 19057709 PMC2592276

[B15] Larsson SC Giovannucci E Wolk A Folate and risk of breast cancer: a meta-analysis J Natl Cancer Inst 2007 99 1 64 76 10.1093/jnci/djk006 17202114

[B16] Kim DH Smith-Warner SA Spiegelman D Yaun SS Colditz GA Freudenheim JL Giovannucci E Goldbohm RA Graham S Harnack L Pooled analyses of 13 prospective cohort studies on folate intake and colon cancer Cancer Causes Control 2010 21 11 1919 1930 10.1007/s10552-010-9620-8 20820900 PMC3082430

[B17] Ma J Stampfer MJ Giovannucci E Artigas C Hunter DJ Fuchs C Willett WC Selhub J Hennekens CH Rozen R Methylenetetrahydrofolate reductase polymorphism, dietary interactions, and risk of colorectal cancer Cancer Res 1997 57 6 1098 1102 9067278

[B18] Kim YI Nutritional epigenetics: impact of folate deficiency on DNA methylation and colon cancer susceptibility J Nutr 2005 135 11 2703 2709 16251634 10.1093/jn/135.11.2703

[B19] Choi SW Friso S Keyes MK Mason JB Folate supplementation increases genomic DNA methylation in the liver of elder rats Br J Nutr 2005 93 1 31 35 10.1079/BJN20041283 15705222

[B20] Pradhan S Bacolla A Wells RD Roberts RJ Recombinant human DNA (cytosine-5) methyltransferase. I. Expression, purification, and comparison of de novo and maintenance methylation J Biol Chem 1999 274 46 33002 33010 10.1074/jbc.274.46.33002 10551868

[B21] Zucker KE Riggs AD Smith SS Purification of human DNA (cytosine-5-)-methyltransferase J Cell Biochem 1985 29 4 337 349 10.1002/jcb.240290407 4086509

[B22] Okano M Bell DW Haber DA Li E DNA methyltransferases Dnmt3a and Dnmt3b are essential for de novo methylation and mammalian development Cell 1999 99 3 247 257 10.1016/S0092-8674(00)81656-6 10555141

[B23] Lyko F Ramsahoye BH Kashevsky H Tudor M Mastrangelo MA Orr-Weaver TL Jaenisch R Mammalian (cytosine-5) methyltransferases cause genomic DNA methylation and lethality in Drosophila Nat Genet 1999 23 3 363 366 10.1038/15551 10545955

[B24] Liang G Chan MF Tomigahara Y Tsai YC Gonzales FA Li E Laird PW Jones PA Cooperativity between DNA methyltransferases in the maintenance methylation of repetitive elements Mol Cell Biol 2002 22 2 480 491 10.1128/MCB.22.2.480-491.2002 11756544 PMC139739

[B25] Chen T Ueda Y Dodge JE Wang Z Li E Establishment and maintenance of genomic methylation patterns in mouse embryonic stem cells by Dnmt3a and Dnmt3b Mol Cell Biol 2003 23 16 5594 5605 10.1128/MCB.23.16.5594-5605.2003 12897133 PMC166327

[B26] Saw SM Yuan JM Ong CN Arakawa K Lee HP Coetzee GA Yu MC Genetic, dietary, and other lifestyle determinants of plasma homocysteine concentrations in middle-aged and older Chinese men and women in Singapore Am J Clin Nutr 2001 73 2 232 239 11157318 10.1093/ajcn/73.2.232

[B27] Chun JY Bae JS Park TJ Kim JY Park BL Cheong HS Lee HS Kim YJ Shin HD Putative association of DNA methyltransferase 1 (DNMT1) polymorphisms with clearance of HBV infection BMB Rep 2009 42 12 834 839 10.5483/BMBRep.2009.42.12.834 20044957

[B28] Fan H Liu D Qiu X Qiao F Wu Q Su X Zhang F Song Y Zhao Z Xie W A functional polymorphism in the DNA methyltransferase-3A promoter modifies the susceptibility in gastric cancer but not in esophageal carcinoma BMC Med 2010 8 12 20 10.1186/1741-7015-8-12 20128888 PMC2829483

[B29] Yang AS Estecio MR Doshi K Kondo Y Tajara EH Issa JP A simple method for estimating global DNA methylation using bisulfite PCR of repetitive DNA elements Nucleic Acids Res 2004 32 3 e38 10.1093/nar/gnh032 14973332 PMC373427

[B30] Singer H Walier M Nusgen N Meesters C Schreiner F Woelfle J Fimmers R Wienker T Kalscheuer VM Becker T Methylation of L1Hs promoters is lower on the inactive X, has a tendency of being higher on autosomes in smaller genomes and shows inter-individual variability at some loci Hum Mol Genet 2011 21 1 219 235 21972244 10.1093/hmg/ddr456 PMC3235015

[B31] Zhang FF Cardarelli R Carroll J Fulda KG Kaur M Gonzalez K Vishwanatha JK Santella RM Morabia A Significant differences in global genomic DNA methylation by gender and race/ethnicity in peripheral blood Epigenetics 2011 6 5 623 629 10.4161/epi.6.5.15335 21739720 PMC3230547

[B32] El-Maarri O Walier M Behne F Van Uum J Singer H Diaz-Lacava A Nusgen N Niemann B Watzka M Reinsberg J Methylation at global LINE-1 repeats in human blood are affected by gender but not by age or natural hormone cycles PLoS One 2011 6 1 e16252 10.1371/journal.pone.0016252 21311577 PMC3023801

[B33] Loehrer FM Angst CP Brunner FP Haefeli WE Fowler B Evidence for disturbed *S*-adenosylmethionine : *S*-adenosylhomocysteine ratio in patients with end-stage renal failure: a cause for disturbed methylation reactions? Nephrol Dial Transplant 1998 13 3 656 661 10.1093/ndt/13.3.656 9550643

[B34] Stabler SP Allen RH Dolce ET Johnson MA Elevated serum *S*-adenosylhomocysteine in cobalamin-deficient elderly and response to treatment Am J Clin Nutr 2006 84 6 1422 1429 17158426 10.1093/ajcn/84.6.1422

[B35] Inoue-Choi M Nelson HH Robien K Arning A Bottiglieri T Koh W-P Yuan J-M One-carbon metabolism nutrient status and plasma *S*-adenosylmethionine concentrations in middle-aged and older Chinese in Singapore Int J Mol Epidemiol Genet 2012 3 2 160 173 22724053 PMC3376917

[B36] Yi P Melnyk S Pogribna M Pogribny IP Hine RJ James SJ Increase in plasma homocysteine associated with parallel increases in plasma *S*-adenosylhomocysteine and lymphocyte DNA hypomethylation J Biol Chem 2000 275 38 29318 29323 10.1074/jbc.M002725200 10884384

[B37] Castro R Rivera I Martins C Struys EA Jansen EE Clode N Graca LM Blom HJ Jakobs C De Almeida IT Intracellular *S*-adenosylhomocysteine increased levels are associated with DNA hypomethylation in HUVEC J Mol Med (Berl) 2005 83 10 831 836 10.1007/s00109-005-0679-8 15976919

[B38] James SJ Melnyk S Pogribna M Pogribny IP Caudill MA Elevation in *S*-adenosylhomocysteine and DNA hypomethylation: potential epigenetic mechanism for homocysteine-related pathology J Nutr 2002 132 8 Suppl 2361S 2366S 12163693 10.1093/jn/132.8.2361S

[B39] Gaudet F Hodgson JG Eden A Jackson-Grusby L Dausman J Gray JW Leonhardt H Jaenisch R Induction of tumors in mice by genomic hypomethylation Science 2003 300 5618 489 492 10.1126/science.1083558 12702876

[B40] Eden A Gaudet F Waghmare A Jaenisch R Chromosomal instability and tumors promoted by DNA hypomethylation Science 2003 300 5618 455 10.1126/science.1083557 12702868

[B41] Okano M Xie S Li E Cloning and characterization of a family of novel mammalian DNA (cytosine-5) methyltransferases Nat Genet 1998 19 3 219 220 10.1038/890 9662389

[B42] Lyon MF LINE-1 elements and X chromosome inactivation: a function for "junk" DNA? Proc Natl Acad Sci U S A 2000 97 12 6248 6249 10.1073/pnas.97.12.6248 10841528 PMC33995

[B43] Bailey JA Carrel L Chakravarti A Eichler EE Molecular evidence for a relationship between LINE-1 elements and X chromosome inactivation: the Lyon repeat hypothesis Proc Natl Acad Sci U S A 2000 97 12 6634 6639 10.1073/pnas.97.12.6634 10841562 PMC18684

[B44] Van Driel LM Eijkemans MJ De Jonge R De Vries JH Van Meurs JB Steegers EA Steegers-Theunissen RP Body mass index is an important determinant of methylation biomarkers in women of reproductive ages J Nutr 2009 139 12 2315 2321 10.3945/jn.109.109710 19812220

[B45] Kerins DM Koury MJ Capdevila A Rana S Wagner C Plasma *S*-adenosylhomocysteine is a more sensitive indicator of cardiovascular disease than plasma homocysteine Am J Clin Nutr 2001 74 6 723 729 11722952 10.1093/ajcn/74.6.723

[B46] Loehrer FM Tschopl M Angst CP Litynski P Jager K Fowler B Haefeli WE Disturbed ratio of erythrocyte and plasma *S*-adenosylmethionine/*S*-adenosylhomocysteine in peripheral arterial occlusive disease Atherosclerosis 2001 154 1 147 154 10.1016/S0021-9150(00)00449-4 11137094

[B47] Barbosa PR Stabler SP Trentin R Carvalho FR Luchessi AD Hirata RD Hirata MH Allen RH Guerra-Shinohara EM Evaluation of nutritional and genetic determinants of total homocysteine, methylmalonic acid and *S-*adenosylmethionine/*S*-adenosylhomocysteine values in Brazilian childbearing-age women Clin Chim Acta 2008 388 1–2 139 147 18023275 10.1016/j.cca.2007.10.023

[B48] Struys EA Jansen EE De Meer K Jakobs C Determination of *S*-adenosylmethionine and *S*-adenosylhomocysteine in plasma and cerebrospinal fluid by stable-isotope dilution tandem mass spectrometry Clin Chem 2000 46 10 1650 1656 11017945

[B49] Melnyk S Pogribna M Pogribny IP Yi P James SJ Measurement of plasma and intracellular *S-*adenosylmethionine and *S*-adenosylhomocysteine utilizing coulometric electrochemical detection: alterations with plasma homocysteine and pyridoxal 5'-phosphate concentrations Clin Chem 2000 46 2 265 272 10657384

[B50] Gellekink H Van Oppenraaij-Emmerzaal D Van Rooij A Struys EA Den Heijer M Blom HJ Stable-isotope dilution liquid chromatography-electrospray injection tandem mass spectrometry method for fast, selective measurement of *S*-adenosylmethionine and *S*-adenosylhomocysteine in plasma Clin Chem 2005 51 8 1487 1492 10.1373/clinchem.2004.046995 15919880

[B51] Lander ES Linton LM Birren B Nusbaum C Zody MC Baldwin J Devon K Dewar K Doyle M FitzHugh W Initial sequencing and analysis of the human genome Nature 2001 409 6822 860 921 10.1038/35057062 11237011

[B52] Venter JC Adams MD Myers EW Li PW Mural RJ Sutton GG Smith HO Yandell M Evans CA Holt RA The sequence of the human genome Science 2001 291 5507 1304 1351 10.1126/science.1058040 11181995

